# Antibody Persistence and Risk of COVID-19 Infection: Insights from Modeling

**DOI:** 10.3390/vaccines12091079

**Published:** 2024-09-21

**Authors:** Laurent Coudeville, Eleine Konate, Tabassome Simon, Xavier de Lamballerie, Scott Patterson, Clotilde El Guerche-Séblain, Odile Launay

**Affiliations:** 1 Global Medical, Vaccines, 69007 Sanofi Lyon, France; laurent.coudeville@sanofi.com; 2 Assistance Publique Hôpitaux de Paris (APHP), Hôpital Cochin, CIC Cochin Pasteur, Inserm, 75014 Paris, France; 3 Sorbonne Université, Department of Clinical Pharmacology and Clinical Research Platform (URC-CRC-CRB), 75012 Paris, France; 4 Aix-Marseille University, IRD-Inserm-IRBA, 13005 Marseille, France; 5 Biostatistics Department, Sanofi Vaccines, Swiftwater, PA 18370, USA; 6 Université Paris Cité; Inserm, I-REIVAC, French Clinical Research Infrastructure Network (F-CRIN), 75014 Paris, France

**Keywords:** antibody persistence, statistical modeling, COVID-19, correlates of protection, neutralizing antibody titer

## Abstract

Background: In this post hoc exploratory study of the APHP-COVIBOOST trial (NCT05124171), we used statistical modeling to describe the evolution of neutralizing antibody (nAb) titers over time, asses its impact on SARS-CoV-2 infection, and explore potential differences between three booster vaccine formulations (D614, B.1.351, and BNT162b2). Methods: Antibody titers were measured for 208 adult participants at day 28, 3 months, and 6 months using a microneutralization assay against different Omicron subvariants. We developed four specific Bayesian statistical models based on a core model, accounting for vaccine-specific antibody decline, boosting of nAb for breakthrough infection, and risk of infection according to nAb levels. The model findings were cross-verified using another validated microneutralization assay. Results: The decrease in nAb titers was significantly lower for the B.1.351 vaccine than for the other booster formulations. An inverse relationship was found between risk of infection upon exposure and nAb levels. With Omicron BA.1 data, these results translated into a positive relative vaccine efficacy against any infection over 6 months for the B.1.351 vaccine compared to the BNT162b2 (31%) and D614 (21%) vaccines. Conclusions: Risk of infection decreased with increasing nAb titers for all vaccines. Statistical models predicted significantly better antibody persistence for the B.1.351 booster formulation compared to the other evaluated vaccines.

## 1. Introduction

As of December 2023, severe acute respiratory syndrome coronavirus 2 (SARS-CoV-2) has caused over 6.9 million deaths with more than 772 million confirmed cases across the globe [1,2]. Although the World Health Organization (WHO) ended the public health emergency of international concern for coronavirus disease (COVID-19) in May 2023, governments are recommended to remain vigilant and maintain the established COVID-19 infrastructure [3]. The major challenges during the COVID-19 pandemic were the rapid emergence of novel SARS-CoV-2 variants with immune escape capacity and waning of vaccine-derived protection [4,5].

Furthermore, another related challenge has been identifying correlates of protection (CoPs), which are immune markers that can be reliably deployed to assess the efficacy of a vaccine [6]. An established CoP is often used as the primary endpoint in the approval of vaccines. However, establishing a CoP is a rigorous process involving evidence generation from multiple sources such as natural history studies, efficacy trials, and mechanistic studies, which in this case is further complicated by the rapid emergence of novel SARS-CoV-2 variants [6,7]. Therefore, although a number of recent studies have accumulated strong evidence that the anti-SARS-CoV-2 neutralizing antibody (nAb) titer could be a potential CoP for COVID-19 vaccines [6,8,9,10,11], wide acceptance of this titer as a CoP for COVID-19 vaccines is yet to be achieved [6].

We have recently published the results of a multicenter, randomized, single-blind trial comparing safety and immunogenicity 15 days after administration of a monovalent ancestral strain recombinant protein vaccine (Sanofi-GSK MVD614, Lyon, France), a monovalent beta variant recombinant protein vaccine (Sanofi-GSK MVB.1.351, VidPrevtyn Beta, Lyon, France), and a monovalent ancestral strain mRNA vaccine (BNT162b2, Pfizer-BioNTech, Mainz, Germany). The results showed that the B.1.351 vaccine produced higher nAb against a wide range of variants (ancestral strain, Beta, Delta, Omicron BA.1) [12]. Subsequently, 3 months (M3) and 6 months (M6) post-booster data also showed that the beta variant recombinant protein vaccine induced higher and durable cross-neutralizing antibodies against Omicron subvariants [13,14]. In the present post hoc exploratory analysis, we utilized these immunogenicity data along with observations of the number of infections to construct a Bayesian model with three objectives: (i) to investigate the evolution of nAb titers over time, (ii) to assess the relationship between nAb and infection, and (iii) to explore the potential differences between different vaccine booster formulations [6]. The outcomes of this analysis will provide insights into the interplay between antibody persistence and protection against infection over time and will contribute to the existing pool of knowledge on CoPs for COVID-19.

## 2. Methods

### 2.1. Study DATA

#### 2.1.1. Study Population

The present study describes a post hoc exploratory analysis based on the results of APHP-COVIBOOST trial, a randomized, single-blinded multicenter clinical trial (ClinicalTrials.gov number, NCT05124171; EudraCT number, 2021-004550-33) [12]. Data from 208 adult participants previously primed with two doses of BNT162b2 and corresponding to the original per-protocol population at day 28 (D28) post vaccination were included in this analysis [12]. Participants were randomized into three groups based on the booster vaccine formulation received at baseline: Sanofi-GSK MVB.1.351 (B.1.351) (*n* = 65), Sanofi-GSK MVD614 (D614) (*n* = 73), and Pfizer-BioNTech BNT162b2 (BNT162b2) (*n* = 70). All data were collected between January and July 2022.

#### 2.1.2. Immunological Assays

A virus microneutralization assay, previously tested for both Zika and SARS-CoV-2 viruses [15,16], was used for the detection of nAb at Aix-Marseille University Laboratories (Marseille, France). Although nAb titers were measured for different subvariants of Omicron in the COVIBOOST trial [12], only data for the Omicron BA.1 strain that was in circulation during the study period were used for modeling. In addition, the nAb titers against Omicron BA.4/5 strain data from the validated pseudovirus microneutralization assay (Monogram Biosciences, South San Francisco, CA, USA) were used as a part of the post hoc analysis, in order to cross-validate the model findings [17].

#### 2.1.3. COVID-19 Detection

All participants were screened for COVID-19 on day 0 (D0) to meet the inclusion criteria. In this post hoc analysis, both symptomatic and asymptomatic COVID-19 infections were identified between D28 and M6 by testing the participants at D28, M3, and M6, or whenever they developed clinical symptoms. Positive cases were defined as having a positive test (RT-PCR or serology), anti-nucleocapsid protein serology, or a 4-fold or higher increase in nAb titers against the corresponding strains.

### 2.2. Statistical Analyses

#### 2.2.1. Statistical Model

A specific Bayesian statistical model accounting for vaccine-specific antibody decline over time, boosting of nAb in case of breakthrough infection, and risk of infection according to nAb levels was developed for this study. The model is defined below:

(1) $$\left\{\begin{array}{c} X_{i,\;j+1}^{g}\;\sim \;\left\{\begin{array}{c} N\left(f_{g}\left(X_{i,\;j}^{g},\;t_{i,\;j+1}^{g}-t_{i,j}^{g}\right),\;\sigma \right),\;\;\;\;\;\;\;\;\;\;\;\;\;\;\;\;\;if\;I{nf}_{i,j}^{g}=0\\ N\left({(X}_{1,j}^{g}+b),\;\sigma \right),\;\;\;\;\;\;\;\;\;\;\;\;\;\;\;\;\;\;\;\;\;\;\;\;\;\;\;\;\;\;if\;I{nf}_{i,j}^{g}=1 \end{array}\right.\\ I{nf}_{i,j}^{g}\;\sim \;Bernoulli\left(\pi_{i,j}^{g}\right)\;\;\;\;\;\;\;\;\;\;\;\;\;\;\;\;\;\;\;\;\;\;\;\;\;\;\;\;\;\;\;\;\;\;\;\;\;\;\;\;\;\;\;\;\;\;\;\;\;\;\;\;\;\;\;\;\;\;\;\;\;\;\;\;\;\\ \pi_{i,j}^{g}=1-\exp[-\int_{t_{i,j}^{g}}^{t_{i,j+1}^{g}}\lambda_{j}Risk\left(X\left(t\right)\right)dt]\;\;\;\;\;\;\;\;\;\;\;\;\;\;\;\;\;\;\;\;\;\;\;\;\;\;\;\;\\ X_{i,j}^{g}={\tilde{X}}_{i,j}^{g}+\epsilon_{i,j}\;\;\;\;\;\;\;\;\;\;\;\;\;\;\;\;\;\;\;\;\;\;\;\;\;\;\;\;\;\;\;\;\;\;\;\;\;\;\;\;\;\;\;\;\;\;\;\;\;\;\;\;\;\;\;\;\;\;\;\;\;\;\;\;\;\;\;\;\;\;\;\;\;\;\;\;\;\;\\ \;\epsilon_{i,j}\;\sim \;Uniform\left(-1,\;1\right)\;\;\;\;\;\;\;\;\;\;\;\;\;\;\;\;\;\;\;\;\;\;\;\;\;\;\;\;\;\;\;\;\;\;\;\;\;\;\;\;\;\;\;\;\;\;\;\;\;\;\;\;\;\;\;\;\;\;\;\;\;\;\;\;\;\;\; \end{array}\right.\;\;\;\;\;\;\;\;\;\;\;\;\;\;\;\;\;\;\;\;\;\;\;$$

where:

$t_{i,j}^{g}:$ Time at which data point *j* for subject *i* in group *g* is observed.

$X_{i,j}^{g}:$ Actual log-transformed titer for subject *i* in group *g* at data point *j*.

${\tilde{X}}_{i,j}^{g}:$ Observed log-transformed titer for subject *i* in group g at data point *j*.

${Inf}_{i,j}^{g}:$ Infection observed between time *t^g^_i,j_* and time *t^g^_i,j+1_*.

$\epsilon_{i,j}$: Titer measurement error.

b: Boosting effect in case of breakthrough infection.

σ: Standard deviation of the antibody persistence model.

$\pi_{i,j}^{g}:$ Probability of infection between time *t^g^_i,j_* and time *t^g^_i,j+1_*.

$\lambda_{j}:$ Force of infection during the period between data point *j* and data point *j* + 1, i.e., rate at which susceptible individuals acquire infection per unit time [18].

$Risk\left(X\right):$ Level of risk of infection for given titer.

$f_{g}\left(X,t\right):$ Group-specific antibody decline as a function of the previously measured titer and time between measurements.

Although antibody persistence was specific to each study arm in this model, the relationship between antibody titers and protection against infection was common to all age groups. All estimations were performed based on the observed data on antibody titers (*X*) and occurrence of infection (${Inf}_{i,j}^{g}$) between data points. The model used log-transformed antibody titers, i.e., an increase in X by 1 corresponds to an increase in titer dilution and an increase by 0 corresponds to subjects with no detectable titers.

Based on the general model described above, four specific models were considered with varying antibody persistence functions (*f_g_*(*X*,*t*)) and levels of risk (*Risk*(*X*)) for a given titer. Model 1 consisted of a linear antibody decline function along with a logistic curve for the evolution of protection with titers. For this model:

(2) $$f_{g}\left(X,t\right)=X-d_{g}t$$

(3) $$R\left(X\right)=1-\frac{1}{1+e^{-(X-m)/s}}$$

(4) $$Risk\left(X\right)=R\left(X\right)/R(0)$$

where *m* and *s* are the location parameter and scale parameter of the logistic function, respectively, and *d_g_* is the antibody waning rate for the group *g*. For Model 1 and all other models, the level of risk obtained is normalized to 1 for participants with no detectable titers, i.e., *Risk*(0) = 1.

Model 2 is based on an exponential antibody decline function and the same logistic curve for the evolution of protection with titer as Model 1.

(5) $$f_{g}\left(X,t\right)=Xe^{-d_{g}t}$$

(6) $$R\left(X\right)=1-\frac{1}{1+e^{-(X-m)/s}}$$

(7) $$Risk\left(X\right)=R\left(X\right)/R(0)$$

Model 3 is based on the same linear antibody decline function as Model 1 and on piecewise linear evolution of protection. Model 3 is defined as

(8) $$f_{g}\left(X,t\right)=X-d_{g}t$$

(9) $$R\left(X\right)=\left\{\begin{array}{c} 1,\;\;\;\;\;\;\;\;\;\;\;\;\;\;\;\;\;\;\;\;\;\;\;\;\;\;\;\;\;\;\;\;\;\;\;\;\;\;\;\;\;\;\;\;if\;X\leq (m-s)\\ \frac{m-X}{s},\;\;\;\;\;\;\;\;\;\;\;\;\;\;\;\;\;\;\;\;\;\;\;\;\;if\;\left(m-s\right)<X<m\\ 0,\;\;\;\;\;\;\;\;\;\;\;\;\;\;\;\;\;\;\;\;\;\;\;\;\;\;\;\;\;\;\;\;\;\;\;\;\;\;\;\;\;\;\;\;\;\;\;\;\;\;\;\;\;\;\;ifX\;\geq m \end{array}\right.$$

(10) $$Risk\left(X\right)=R\left(X\right)/R(0)$$

where *m* is maximum titer for risk of infection (i.e., location parameter), and *s* is the rate of decrease (i.e., scale parameter) in risk according to titer (*Risk* = 1 if *X* = m − s).

Finally, Model 4 is based on the exponential antibody decline function and piecewise linear evolution of protection:

(11) $$\left(X,t\right)=Xe^{-d_{g}t}$$

(12) $$R\left(X\right)=\left\{\begin{array}{c} 1,\;\;\;\;\;\;\;\;\;\;\;\;\;\;\;\;\;\;\;\;\;\;\;\;\;\;\;\;\;\;\;\;\;\;\;\;\;\;\;\;\;\;\;\;if\;X\leq (m-s)\\ \frac{m-X}{s},\;\;\;\;\;\;\;\;\;\;\;\;\;\;\;\;\;\;\;\;\;\;\;\;\;if\;\left(m-s\right)<X<m\\ 0,\;\;\;\;\;\;\;\;\;\;\;\;\;\;\;\;\;\;\;\;\;\;\;\;\;\;\;\;\;\;\;\;\;\;\;\;\;\;\;\;\;\;\;\;\;\;\;\;\;\;\;\;\;\;\;ifX\;\geq m \end{array}\right.$$

(13) $$Risk\left(X\right)=R\left(X\right)/R(0)$$

#### 2.2.2. Model Outcomes

In addition to parameter estimation, statistical model analyses generated a series of outcomes to aid the interpretation of model results. The posterior distributions of model parameters were used to estimate the following outcomes:

Estimated risk of infection according to titer given by the risk function:

(14) Risk(X)

Estimated evolution of antibody titers in the absence of infection by study group in terms of geometric mean titer (GMT), the standard metric for population-level immunogenicity data [12,14]:

(15) $${GMT}_{j}^{g}=\frac{1}{n_{g}}\sum_{i=1}^{n_{g}}X_{i,j}^{g}$$

where *n_g_* is the number of subjects in study group *g*.

Relative vaccine efficacy between study groups:

(16) $${RVE}_{a,b}=1-\frac{n_{b}\sum_{j=1}^{T}\sum_{i=1}^{n_{a}}\pi_{i,j}^{a}}{n_{a}\sum_{j=1}^{T}\sum_{i=1}^{n_{b}}\pi_{i,j}^{b}}$$

where *n_a_* and *n_b_* are the number of subjects in study groups a and b (relative efficacy of group a versus group b), respectively, and *T* is the number of periods of observation.

Monthly evolution of protection against infection over time by study arm:

(17) $$Prot_{m}^{g}=1-\frac{\sum_{i=1}^{n_{g}}Risk(f_{g}\left(X_{i,1}^{g},m\right))}{n_{g}}$$

where *m* is number of months since the first observed titer.

#### 2.2.3. Statistical Methods

The model was coded and analyzed using RStan and loo packages in R programming [19,20]. The microneutralization assay data from the Aix-Marseille University laboratories, analyzed using the statistical models, were based on titers against Omicron BA.1 strain, one of the circulating strains during the period of the study. However, relevant data (i.e., titers at D28, M3, and M6) from the Monogram Biosciences laboratory were only available for Omicron BA.4/5 strain and the same were used for analyses.

Non-informative priors were used for parameters related to antibody decline and weakly informative priors for parameters related to risk of infection for a given titer. R-hat convergence diagnostic, effective sample sizes, and Pareto smoothed importance sampling diagnostic plots were used to assess the accuracy and robustness of model fit. Subsequently, expected log pointwise predictive densities (ELPDs), measured using the loo package (elpd_loo), were used to compare the fit estimated by the different models considered [20].

## 3. Results

### 3.1. Immunological Data and Infection Events

nAb titers against Omicron BA.1 strain were measured with the Aix-Marseille assay for all 208 participants at D28, for 205 participants at M3, and for 142 participants at M6 (Table 1) [12,15]. For the Monogram assay (Omicron BA.4/5 strain), the number of data points available was, however, lower: 161, 152, and 118 participants at D28, M3, and M6, respectively.

For the Aix-Marseille assay, a total of 85 infection data points were available for the analysis. Some differences according to vaccine group in the proportion of patients infected were observed: an infection was identified for 23/65 = 35.4% of the subjects included in the B.1.351 group, 32/73 = 43.8% in the D614 group, and 30/70 = 42.8% in the BNT162b2 group. For the Monogram assay, the more limited number of participants resulted in a lower number of available infection data points (71) (Table 2).

### 3.2. Model Selection

Satisfactory convergence was achieved for all four models (Table 3). The force of infection was found to decrease between two consecutive data points (*λ*_1_ vs. *λ*_2_) across the models. Furthermore, all four models predicted the lowest waning of antibody (*d_g_*) for the B.1.351 vaccine group and highest for the BNT162b2 vaccine group.

Model 2 and Model 4 provided a similar level of fit for the Aix-Marseille data and outperformed the other two models, as evident from the ELPD (−1245.4 vs. −1245.2) [20]. The ELPD for Model 4 differed from those for Model 1 and Model 3 by −16.5 and −16.0 units, respectively (Table S1). Thus, Model 2 and Model 4 were considered as the preferred models for the Aix-Marseille assay. Interestingly, leave-one-out (LOO) cross-validation predicted that Model 3 and Model 4 provided the best fit for the Monogram assay data. However, the differences in the levels of fit were limited for the remaining models (Table S2).

### 3.3. Observed vs. Model-Estimated Parameters

**Figure 1:**
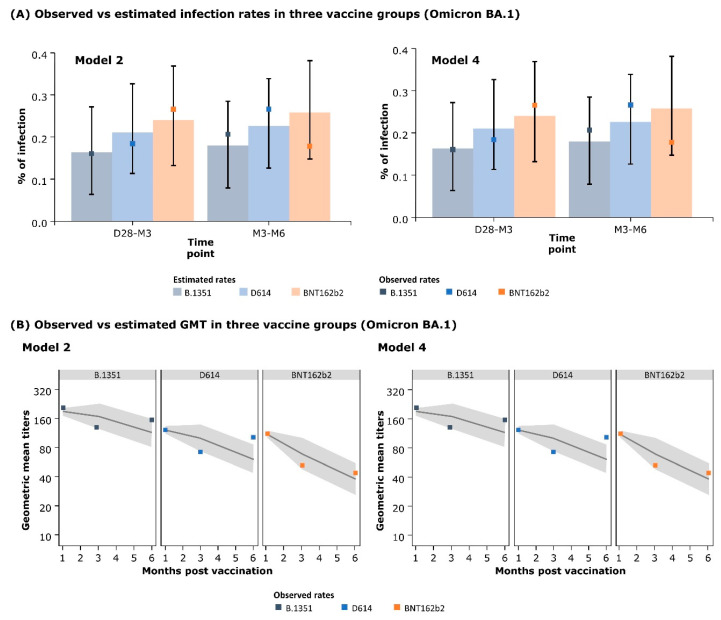
Observed vs. model-estimated parameters for Omicron BA.1 data: (**A**) infection rates and (**B**) geometric mean titers (GMTs) by study arms. The error bars (in Panel **A**) and shaded areas (in Panel **B**) represent 95% credible interval. D28, M3, and M6 denote day 28, month 3, and month 6, respectively.

The model-estimated infection rates and GMT were compared with the observed infection rates and GMT to assess the quality of the models. For the Aix-Marseille data, the observed infection rates in the two time intervals (D28–M3 and M3–M6) lay within the 95% credible interval (CI) of the infection rates estimated by Model 2 and Model 4 (i.e., the preferred models) (Figure 1A). Similarly, the observed GMT also fell between the 95% CI of the estimated GMT, except for the GMT increase between M3 and M6 for the D614 vaccine (Figure 1B). The observed infection rates for the Omicron BA.4/5 strain (Monogram data) lay between the 95% CI of the infection rate estimated by Model 3 (preferred model). The estimated GMT values also accorded with the observed data except for the D614 vaccine group (Figure S1).

### 3.4. Model Outcomes for Aix-Marseille University Data (Omicron BA.1)

Risk of infection vs. microneutralization assay titer (against Omicron BA.1).

**Figure 2:**
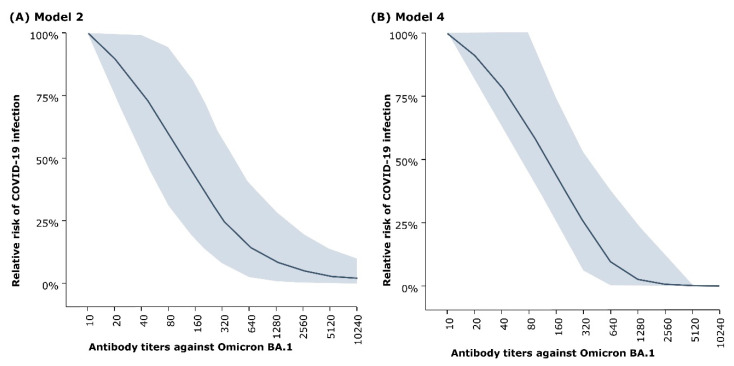
Risk of infection vs. microneutralization assay titer (against Omicron BA.1) estimated by (**A**) Model 2 and (**B**) Model 4. The shaded areas represent 95% credible interval. Logarithmic scale has been used for the X-axes of the graphs to better visualize a long range of data.

The risk curve was obtained from the risk function *Risk(X)* and is presented as the relative risk compared to an individual with no detectable titers. Both Model 2 and Model 4 established a correlation between antibody level and the risk of SARS-CoV-2 infection upon exposure. There was a significant reduction in the risk level with increasing microneutralization titers. Compared to subjects with no detectable antibodies, the relative risk of developing infection upon exposure was <10% for a titer ≥1280 (Figure 2). A similar relationship was also predicted by Model 1 and Model 3 (Figure S2).

#### 3.4.1. Evolution of Antibody Titers (GMT) in the Absence of Infection

**Figure 3:**
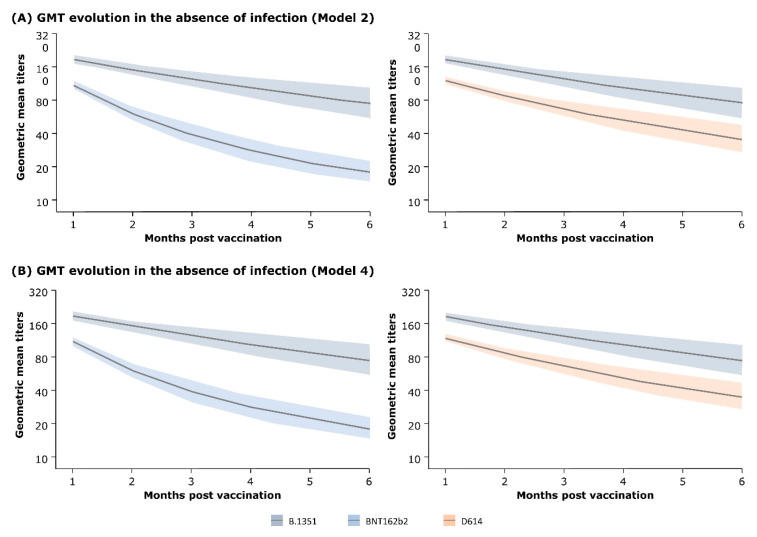
Evolution of antibody titers (GMT) in the absence of infection as estimated by (**A**) Model 2 and (**B**) Model 4 (Omicron BA.1 data). The shaded areas represent 95% credible interval.

In the absence of infection, GMT against Omicron BA.1 steadily declined for all three study groups (i.e., three vaccine groups). The mean GMT estimated by Model 2 decreased from 188.3 at month 1 (M1) to 75.6 at month 6 (M6) for the B.1.351 vaccine group. The changes in the mean GMT of the D614 and BNT162b2 vaccine groups were 120.9 to 35.2 and 110.3 to 18.0, respectively. Similar changes in GMT values were predicted by Model 4. The rate of decrease in GMT was significantly lower for the B.1.351 vaccine than for the other vaccines (Figure 3).

#### 3.4.2. Evolution of Protection against Infection over Time by Study Arm (against Omicron BA.1)

**Figure 4:**
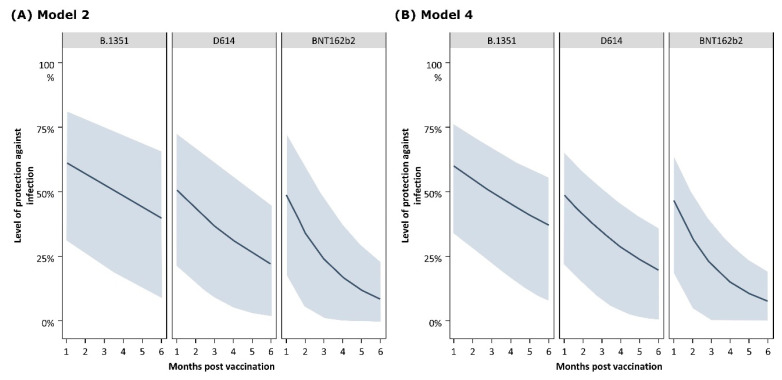
Evolution of protection against infection over time (monthly) by study arm (against Omicron BA.1) estimated by (**A**) Model 2 and (**B**) Model 4. The shaded areas represent 95% credible interval.

The results from Model 2 indicate that in the absence of reinfection, for the B.1.351 vaccine, protection against infection is maintained for at least 6 months at a higher level (39.9% [9.0–65.2]) as compared to the D614 (22.1% [95% CI: 2.0–44.4]) and BNT162b2 (8.8% [95% CI: 0.1–22.9]) vaccines. (Figure 4). A similar trend was also predicted by Model 4. However, the differences between the vaccine groups were not significant considering the large CIs.

#### 3.4.3. Relative Vaccine Efficacy between Study Groups (against Omicron BA.1)

The relative efficacy of the B.1.351 vaccine is presented against those of the D614 and BNT162b2 vaccines. This efficacy was calculated using the expected evolution of titers and corresponding probability of infection over the 6 months for which observed data were obtained. As predicted by the antibody persistence and evolution of protection curves, the better protection conferred by the B.1.351 vaccine translated into a significantly positive relative efficacy of the B.1.351 vaccine as compared to those of the BNT162b2 (31% [95% CI: 15–45]) and D614 (21% [95% CI: 10–32]) vaccines. Model 4 also predicted a similar relative vaccine efficacy of the B.1.351 vaccine against those of the BNT162b2 (30% [95% CI: 17–41]) and D614 (21% [95% CI: 11–31]) vaccines.

### 3.5. Model Outcomes for Monogram Biosciences Data (Omicron BA.4/5)

Analysis of the Monogram assay data (titers against Omicron BA.4/5) with the preferred model (Model 3) showed similar outcomes to those obtained by the Aix-Marseille assay with Omicron BA.1 data (Figure S3). The antibody waning rate was lowest in the B.1.351 vaccine group vs. the D614 and BNT162b2 vaccine groups (0.28 [95% CI: 0.14–0.41], 0.34 [95% CI: 0.21–0.48], and 0.45 [95% CI: 0.31–0.60], respectively). Predicted GMT in the absence of infection remained significantly higher for the B.1.351 vaccine group. At M1, the mean GMTs for the B.1.351, D614, and BNT162b2 groups were predicted to be 824.7, 448.1, and 378.0, which changed to 317.8, 135.9, and 78.3, respectively, at M6. The relative effectiveness of the B.1.351 vaccine was found to be 27% against both the D614 (95% CI: 17–35) and BNT162b2 (95% CI: 17–36) vaccines. Finally, Model 3 predicted that for the B.1.351 vaccine, protection against infection is maintained for at least 6 months at a higher level (37.7% [95% CI: 11.0–62.4]), as compared to the D614 (22.7% [95% CI: 3.5–47.3]) and BNT162b2 (13.1% [95% CI: 0.2–37.8]) vaccines. 

## 4. Discussion

Statistical modeling using real-world clinical data has been a powerful tool in predicting long-term antibody persistence against various pathogens as well as in establishing a relationship between immune markers and vaccine-derived protection [21,22,23]. In the present study, we analyzed clinical data from the APHP-COVIBOOST trial using Bayesian statistical modeling to predict the evolution of nAb titers over time and its relationship with the risk of infection in adults. Adapting the general model, we constructed and compared four specific models to ensure the best possible outcomes for two SARS-CoV-2 subvariants, Omicron BA.1 and BA.4/5. The four models used different mathematical formulations for the antibody decline and risk functions. Despite the differences in the construction of the models and the values of measured parameters, two common tendencies were evident from all four models: (i) the risk of infection is significantly correlated to the microneutralization assay titer, and (ii) compared to the other two study groups, antibody waning was lowest in the recipients of the B.1.351 vaccine.

Of the four models tested, Model 2, which presented an exponential antibody decline function and a logistic curve for the evolution of protection, and Model 4, which presented a linear antibody decline function and piecewise linear evolution of protection function, outperformed the other two models for Omicron BA.1 data and provided the best fit. Despite the differences in the mathematical constitution of the two models, there was remarkable similarity in the relative risk vs. antibody titer curves. The relative risk of infection (with respect to individuals with no nAb titers) decreased with increasing microneutralization assay titers and became very low for individuals with high nAb titers (<10% for titers ≥1280). This finding was consistent with the results of recent studies evaluating the relationship of nAb titers with the risk of symptomatic or asymptomatic SARS-CoV-2 infection, which also predicted a corresponding inverse relationship [6,8,9,10,11]. The projected GMT decreased over time in all three study groups but remained significantly higher in the B.1.351 vaccine group than in the other two vaccine groups. Furthermore, the model showed significantly positive relative vaccine efficacy for the B.1.351 vaccine compared to those for the D614 and BNT162b2 vaccines. Although, the clinical significance of this finding has not yet been established, the result is aligned with the findings of similar studies showing that vaccines conferring higher antibody titers present higher efficacy [9,10]. 

Monogram assay analyses with Omicron BA.4/5 data presented similar trends in outcomes compared to the Aix-Marseille assay analyses with Omicron BA.1 data. These trends included an inversely proportional relationship between the risk level and microneutralization assay titer, a significantly lower decrease in antibody titers over time, and a significantly positive relative vaccine efficacy for the B.1.351 vaccine as compared to the other booster vaccines.

Establishing a CoP for COVID-19 vaccines remains a challenge owing to the rapid emergence of novel SARS-CoV-2 variants [7]. In particular, studies assessing Omicron-specific CoPs are not yet widely reported [7]. Nevertheless, the results of this study showcase the value of modeling data on antibody persistence and vaccine-derived protection and are expected to guide public health considerations and evaluations of new vaccines in the future [24].

However, there are several limitations to this study. First, as the study describes a post hoc exploratory analysis, the case definitions were not predetermined. Second, the results are not transposable to other assays (in terms of technology and variants), and the characteristics of the study period, such as the level of virus circulation and immunity profile of the population, may have impacted the results. In particular, the rapid evolution of subvariants precludes any extrapolation of the current study results. Third, no results were derived for symptomatic cases considering their limited number. Additional limitations include the relatively small sample size, lack of exhaustive model search, and absence of a placebo control group for efficacy evaluation.

## 5. Conclusions

Based on a general Bayesian model, we constructed and used four specific statistical models to predict the antibody persistence and estimate the risk of COVID-19 infection by Omicron BA.1 and BA.4/5 strains. Model analyses revealed an inverse relationship between antibody level and the risk of any asymptomatic or symptomatic SARS-CoV-2 infection upon exposure and estimated a significantly better antibody persistence for the beta-adjuvanted B.1.351 booster vaccine formulation as compared to the other evaluated booster vaccine formulations. These results translated into a positive relative vaccine efficacy against any infection for the B.1.351 booster vaccine formulation as compared to the other booster vaccine formulations. These results highlight the importance of continued data collection for better documentation of the correlation between immune response and protection against COVID-19 for different assays, variants, and outcomes.

## Figures and Tables

**Table 1:** Number of participants with available antibody data.

Dataset	Timepoint	B.1.351	D614	BNT162b2
Aix-Marseille	D28	65	73	70
	M3	63	72	70
	M6 *	48	50	44
Monogram	D28	56	47	58
	M3	53	45	54
	M6 *	42	42	34

* Antibody data at M6 for subjects with infection between D28 and M3 were considered as missing (accounting for possible delay in boosting effect that impacts interpretation of titer evolution between M3 and M6).

**Table 2:** Number of infection events during the D28–M3 and M3–M6 time intervals.

Dataset	Time Interval	B.1.351	D614	BNT162b2
Aix-Marseille	D28–M3	10	13	18
	M3–M6	13	19	12
Monogram	D28–M3	10	1	18
	M3–M6	14	17	11

**Table 3:** Summary results for the four models (Omicron BA.1 data).

Model	Parameter *	Mean	SD	95% CI		n_effective_	Rhat
Model 1	Force of infection, D28–M3 (*λ*_1_) ^†^	0.22	0.07	0.12	0.38	4587	1
	Force of infection, M3–M6 (*λ*_2_) ^†^	0.13	0.03	0.08	0.19	2092	1
	Location parameter of the risk function (*m*)	2.88	1.33	0.3	5.18	2439	1
	Scale parameter of the risk function (*s*)	1.54	0.55	0.48	2.68	932	1.01
	Antibody waning rate for B.1.351 group (*d*_1_)	0.27	0.06	0.15	0.39	2118	1
	Antibody waning rate for D614 group (*d*_2_)	0.39	0.06	0.27	0.5	7932	1
	Antibody waning rate for BNT162b2 (*d*_3_)	0.49	0.06	0.37	0.6	4987	1
	Standard deviation for Ab persistence model (σ)	1.19	0.05	1.1	1.29	4989	1
	Boost in titers post infection (b)	2.08	0.15	1.78	2.37	6911	1
Model 2	Force of infection, D28–M3 (*λ*_1_) ^†^	0.22	0.07	0.12	0.38	5528	1
	Force of infection, M3–M6 (*λ*_2_) ^†^	0.13	0.03	0.08	0.19	5654	1
	Location parameter (*m*)	2.88	1.34	0.33	5.27	3948	1
	Scale parameter (*s*)	1.5	0.54	0.56	2.7	4457	1
	Antibody waning rate for B.1.351 group (*d*_1_)	0.08	0.02	0.04	0.11	7422	1
	Antibody waning rate for D614 group (*d*_2_)	0.14	0.02	0.09	0.18	7289	1
	Antibody waning rate for BNT162b2 (*d*_3_)	0.28	0.04	0.21	0.37	6410	1
	Standard deviation for Ab persistence model (σ)	1.19	0.04	1.1	1.28	6038	1
	Boost in titers post infection (b)	2.08	0.15	1.78	2.38	8932	1
Model 3	Force of infection, D28–M3 (*λ*_1_) ^†^	0.21	0.05	0.12	0.31	7529	1
	Force of infection, M3–M6 (*λ*_2_) ^†^	0.12	0.02	0.08	0.17	8535	1
	Location parameter of the risk function (*m*)	6.68	0.92	5.48	8.92	5586	1
	Scale parameter of the risk function (*s*)	7	2.64	2.89	13.3	5334	1
	Antibody waning rate for B.1.351 group (*d*_1_)	0.27	0.06	0.15	0.38	8715	1
	Antibody waning rate for D614 group (*d*_2_)	0.39	0.06	0.27	0.5	9967	1
	Antibody waning rate for BNT162b2 (*d*_3_)	0.49	0.06	0.37	0.6	8858	1
	Standard deviation for Ab persistence model (σ)	1.19	0.05	1.1	1.29	6096	1
	Boost in titers post infection (b)	2.08	0.15	1.77	2.37	12,295	1
Model 4	Force of infection, D28–M3 (*λ*_1_) ^†^	0.21	0.05	0.12	0.31	6704	1
	Force of infection, M3–M6 (*λ*_2_) ^†^	0.12	0.02	0.08	0.17	7841	1
	Location parameter of the risk function (*m*)	6.52	0.97	5.26	9.04	5616	1
	Scale parameter of the risk function (*s*)	6.86	2.69	2.77	13.11	5524	1
	Antibody waning rate for B.1.351 group (*d*_1_)	0.08	0.02	0.04	0.11	7818	1
	Antibody waning rate for D614 group (*d*_2_)	0.14	0.02	0.09	0.18	7867	1
	Antibody waning rate for BNT162b2 (*d*_3_)	0.28	0.04	0.21	0.37	7574	1
	Standard deviation for Ab persistence model (σ)	1.19	0.05	1.1	1.28	6821	1
	Boost in titers post infection (b)	2.08	0.15	1.78	2.38	10535	1

Ab, antibody; CI, credible interval; SD, standard deviation. * The measured parameters were common in all three models, but the physical interpretations differed for *m* and *s*. ^†^ Force of infection refers to the rate at which susceptible individuals acquire infection per unit time.

## Data Availability

The datasets generated and/or analyzed during the current study are not publicly available but are available from the corresponding author on reasonable request.

## Supplementary Materials

The following supporting information can be downloaded at: https://www.mdpi.com/article/10.3390/vaccines12091079/s1, Figure S1: Observed vs model-estimated parameters for Omicron BA.4/5 data: (A) infection rates and (B) GMT by study arms; Figure S2: Risk of infection vs microneutralization assay titer (against Omicron BA.1) estimated by (A) Model 1 and (B) Model 3; Figure S3: Outcomes of Model 3 (Omicron BA.4/5) based on the Monogram assay data: (A) Risk of infection vs microneutralization assay titer. (B) Evolution of antibody titers (GMT) in the absence of infection. (C) Evolution of protection against infection over time (monthly) by study arm; Table S1: Summary of leave-one-out cross validation analysis and Pareto k diagnostics for the four models (Omicron BA.1 data); Table S2: Summary of leave-one-out cross validation analysis and Pareto k diagnostics for the four models (Omicron BA.4/5 data).

## Abbreviation

CI, credible interval; CoP, correlate of protection; COVID-19, coronavirus disease; ELPD, expected log pointwise predictive density; GMT, geometric mean titer; nAb, neutralizing antibody; SARS-CoV-2, severe acute respiratory syndrome coronavirus 2; SD, standard deviation; WHO, World Health Organization.
