# Supplementary material for: Antibody Persistence and Risk of COVID-19 Infection: Insights from Modeling
Source: Vaccines (Basel). 2024 Sep 21;12(9):1079. doi: 10.3390/vaccines12091079 (PMC11435888; doi:10.3390/vaccines12091079)
Supplement: Supplementary file 1 [file vaccines-12-01079-s001.zip › vaccines-3168425-supplementary.pdf]

Antibody Persistence and Risk of COVID-19 Infection: Insights from Modeling

**Laurent Coudeville [1,†], Eleine Konate [2,†], Tabassome Simon [3], Xavier de Lamballerie [4], Scott Patterson [5],
Clotilde El Guerche-Séblain [1,*] and Odile Launay [2,6,*] on behalf of AP-PH COVIBOOST Vaccine Trial Group**

SUPPLEMENTARY MATERIAL

Figure S1. Observed vs model-estimated parameters for Omicron BA.4/5 data: (A) infection rates and (B) GMT by study arms.

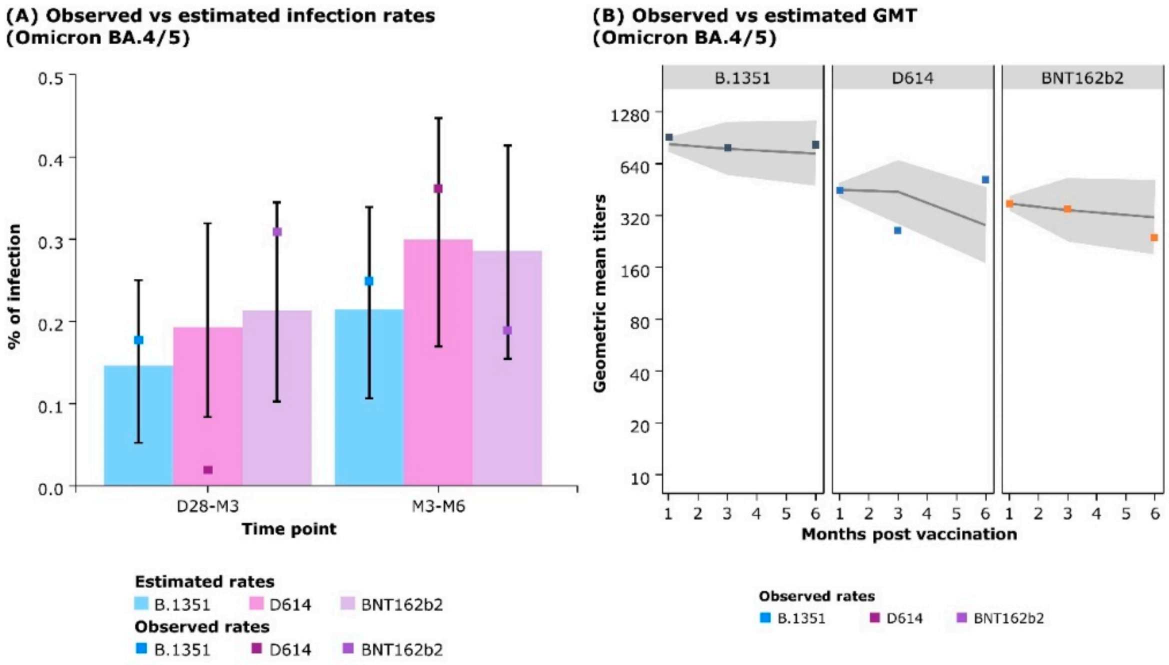

The error bars (in Panel A) and shaded areas (in Panel B) represent 95% credible interval. D28, M3, and M6 denotes day 28, month 3, and month 6, respectively.

Figure S2. Risk of infection vs microneutralization assay titer (against Omicron BA.1) estimated by (A) Model 1 and (B) Model 3.

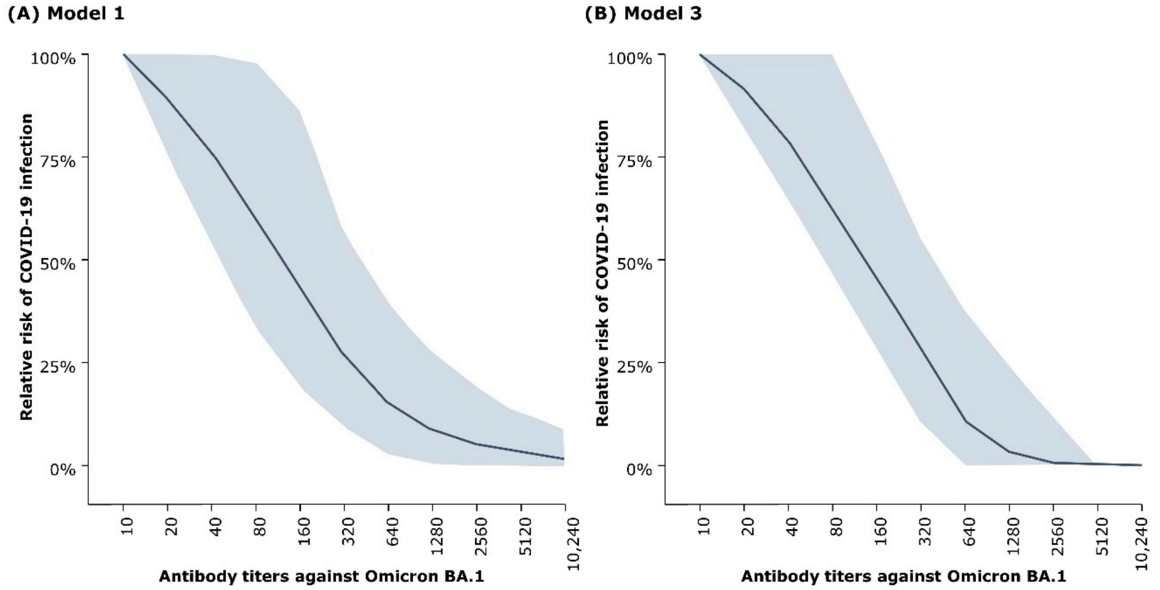

The shaded areas represent 95% credible interval. Logarithmic scale has been used for the X-axes of the graphs to better visualize a long range of data.

Figure S3. Outcomes of Model 3 (Omicron BA.4/5) based on the Monogram assay data: (A) Risk of infection vs microneutralization assay titer. (B) Evolution of antibody titers (GMT) in the absence of infection. (C) Evolution of protection against infection over time (monthly) by study arm.

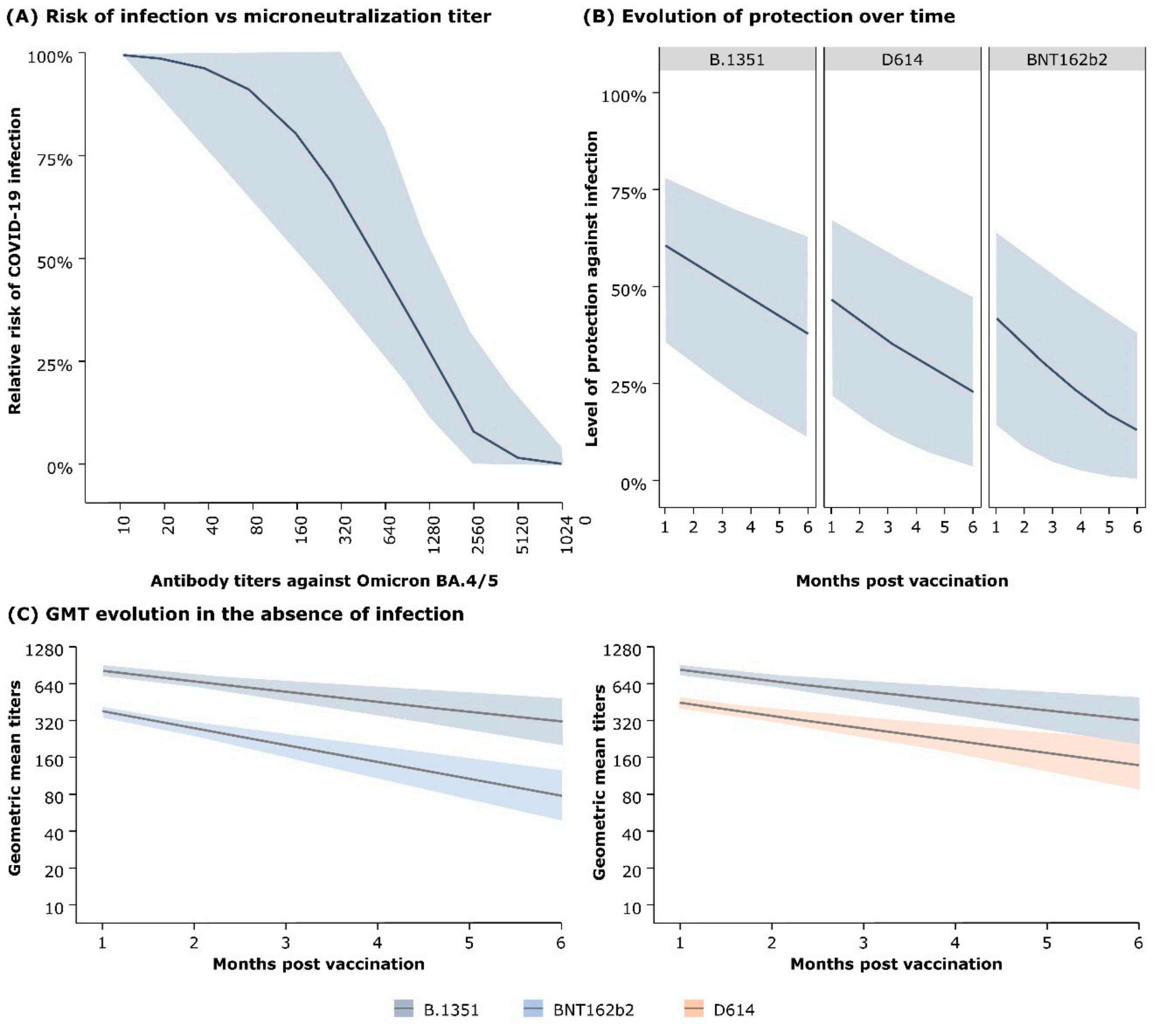

The shaded areas represent 95% credible interval

Table S1. Summary of leave-one-out cross validation analysis and Pareto k diagnostics for the four models (Omicron BA.1 data).

Parameter	Model 1	Model 2	Model 3	Model 4
ELPD	−1261.7 ± 20.8	−1245.4 ± 21.0	−1261.2 ± 20.6	−1245.2 ± 20.8
p_loo	143.2 ± 6.4	126.2 ± 6.3	143.0 ± 6.4	126.0 ± 6.2
Information criteria (looic)	2523.3 ± 41.5	2490.8 ± 42.1	2522.4 ± 41.3	2490.4 ± 41.7
$-\infty < k \leq 0.5$	122 (58.7%)	159 (76.4%)	119 (57.2%)	149 (71.6%)
$0.5 \leq k \leq 0.7$	62 (29.8%)	31 (14.9%)	65 (31.2%)	36 (17.3%)
$0.7 \leq k \leq 1$	23 (11.1%)	18 (8.7%)	22 (10.6%)	21 (10.1%)
$1 \leq k < \infty$	1 (0.5%)	0 (0%)	2 (1.0%)	2 (1.0%)

ELPD, expected log pointwise predictive density; k, pareto k value; p_loo, effective number of parameters and can be computed as the difference between ELPD and the non-cross-validated log posterior predictive density.

Table S2. Summary of leave-one-out cross validation analysis and Pareto k diagnostics for the four models (Omicron BA.4/5 data).

Parameter	Model 1	Model 2	Model 3	Model 4
ELPD	−993.4 ± 21.0	−995.3 ± 20.5	−990.8 ± 20.8	−992.5 ± 20.2
p_loo	88.5 ± 5.5	81.6 ± 5.3	87.4 ± 5.4	80.1 ± 5.0
Information criteria (looic)	1986.9 ± 41.9	1990.7 ± 41.0	1981.7 ± 41.7	1985.0 ± 40.4
$-\infty < k \leq 0.5$	123 (76.4%)	129 (80.1%)	121 (75.2%)	129 (80.1%)
$0.5 \leq k \leq 0.7$	28 (17.4%)	24 (14.9%)	28 (17.4%)	24 (14.9%)
$0.7 \leq k \leq 1$	10 (6.2%)	8 (5.0%)	12 (7.5%)	8 (5.0%)
$1 \leq k < \infty$	0 (0%)	0 (0%)	0 (0%)	0 (0%)

ELPD, expected log pointwise predictive density; k, pareto k value; p_loo, effective number of parameters and can be computed as the difference between ELPD and the non-cross-validated log posterior predictive density.
